# Supplementary material for: Identification of Freezing-Responsive microRNAs and Their Targets in Chinese Jujube by Small RNA and Degradome Sequencing
Source: Int J Mol Sci. 2026 Sep 4;27(17):7912. doi: 10.3390/ijms27177912 (PMC13566890; doi:10.3390/ijms27177912)
Supplement: Supplementary file 1 [file ijms-27-07912-s001.zip › Supplementary Material S2/novel_miR_165.pdf]

Provisional ID	:	novel_miR_165
Score total	:	0.5
Score for star read(s)	:	-1.3
Score for read counts	:	0
Score for mfe	:	0.2
Score for randfold	:	1.6
Score for cons. seed	:	
Total read count	:	20803
Mature read count	:	20773
Loop read count	:	0
Star read count	:	30

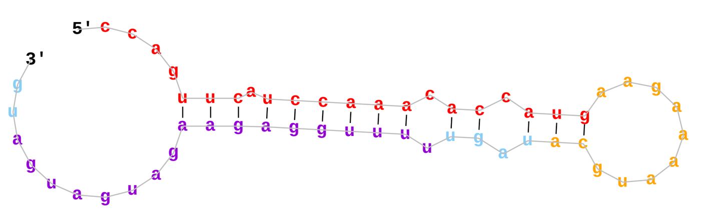

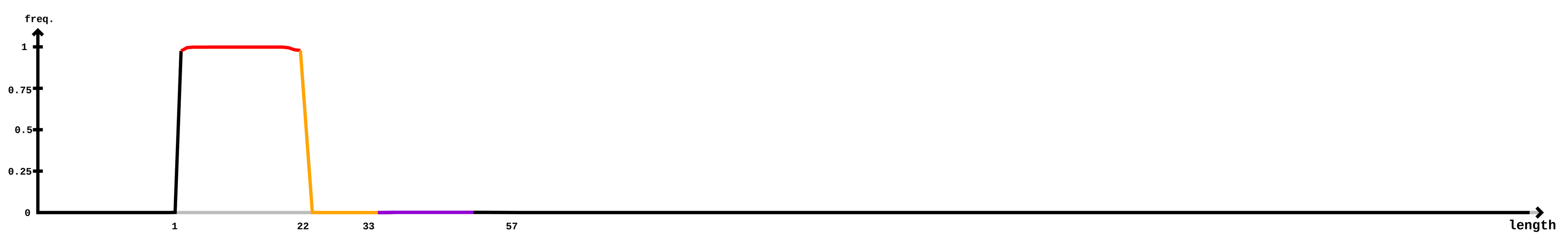

Mature **Star**

```
5'- ucucgacaaucaaggaucuccaccaguucauccaaacaccaugaagaaaaugcauaguuuuuggagaagaugaugauggauaucuuugauccuccaauugcgucaauaagugaaggugaaauuucuucuccccucuugagcuuauugucaaugaaagucucaacuccccuaagacgcaaagcuugguaaagaugcccugugaaaguuuugcguguaucugagccucugaaacucaagaagacaucauauu  -3'  obs
    ucucgacaaucaaggaucuccaccaguucauccaaacaccaugaagaaaaugcauaguuuuuggagaagaugaugauggauaucuuugauccuccaauugcgucaauaagugaaggugaaauuucuucuccccucuugagcuuauugucaaugaaagucucaacuccccuaagacgcaaagcuugguaaagaugcccugugaaaguuuugcguguaucugagccucugaaacucaagaagacaucauauu        exp
    ....((..((((((((.((((.((..((.((((((.((.(((.........))).)).))))))...))...)).)))).)))))))))..)).......(.(((((((((.((.(((.....)))..)).))....)))))))).).((((..((((...(((.....(.((((((((((((((....))))......))))))))))).)....)))..(((........))))))).))))...   reads   mm   sample
    .....................accaguucauccaaacacc..................................................................................................................................................................................................................   1   0   JCK-1
    .....................Cccaguucauccaaacaccaug...............................................................................................................................................................................................................   2   1   JCK-1
    .....................accaguucauccaaacaccaug...............................................................................................................................................................................................................   1   0   JCK-3
    .....................accaguucauccaaacaccaug...............................................................................................................................................................................................................   1   0   JCK-1
    .....................accaguucauccaaacaccaug...............................................................................................................................................................................................................   2   0   JCK-2
    .....................Cccaguucauccaaacaccaug...............................................................................................................................................................................................................   5   1   JCK-3
    .....................Cccaguucauccaaacaccaug...............................................................................................................................................................................................................   7   1   LCK-3
    ......................ccaguucauccaaacacc..................................................................................................................................................................................................................   14   0   JCK-3
    ......................ccaguucauccaaacacc..................................................................................................................................................................................................................   3   0   LT-1
    ......................ccaguucauccaaacacc..................................................................................................................................................................................................................   21   0   LCK-3
    ......................ccaguucauccaaacacc..................................................................................................................................................................................................................   12   0   LT-3
    ......................ccaguucauccaaacacU..................................................................................................................................................................................................................   1   1   LT-3
    ......................ccaguucauccaaacacc..................................................................................................................................................................................................................   9   0   JCK-2
    ......................ccaguucauccaaacacU..................................................................................................................................................................................................................   2   1   JCK-2
    ......................ccaguucauccaaacacc..................................................................................................................................................................................................................   12   0   JCK-1
    ......................ccaguucauccaaacacU..................................................................................................................................................................................................................   3   1   LT-2
    ......................ccaguucauccaaacacc..................................................................................................................................................................................................................   2   0   LT-2
    ......................ccaguucauccaaacacca.................................................................................................................................................................................................................   8   0   LT-1
    ......................ccaguucauccaaaNacca.................................................................................................................................................................................................................   1   1   LCK-1
    ......................ccaguucauccaaacacca.................................................................................................................................................................................................................   10   0   LT-3
    ......................ccaguucauccaaacacca.................................................................................................................................................................................................................   5   0   JT-3
    ......................ccaguucauccaaacacca.................................................................................................................................................................................................................   33   0   JCK-1
    ......................ccaguucauccaaacaccU.................................................................................................................................................................................................................   1   1   JCK-3
    ......................ccaguucauccaaacacca.................................................................................................................................................................................................................   2   0   JT-2
    ......................ccaguucauccaCacacca.................................................................................................................................................................................................................   1   1   JCK-3
    ......................ccaguucauccaaacacca.................................................................................................................................................................................................................   30   0   JCK-3
    ......................ccaguucauccaaacacca.................................................................................................................................................................................................................   34   0   LCK-1
    ......................ccCguucauccaaacacca.................................................................................................................................................................................................................   1   1   LT-2
    ......................ccaguucauccaaacacca.................................................................................................................................................................................................................   23   0   LCK-2
    ......................ccaguucauccaaacacca.................................................................................................................................................................................................................   16   0   LCK-3
    ......................ccaguucauccaaacacca.................................................................................................................................................................................................................   45   0   JCK-2
    ......................ccaguucauccaaacacca.................................................................................................................................................................................................................   8   0   LT-2
    ......................ccaguucauccaaacacUa.................................................................................................................................................................................................................   1   1   JCK-2
```

```
                      Mature                       Star
ucucgacaaucaaggaucuccaccaguucauccaaacaccaugaagaaaaugcauaguuuuuggagaagaugaugauggauaucuuugauccuccaauugcgucaauaagugaaggugaaauuucuucuccccucuugagcuuauugucaaugaaagucucaacuccccuaagacgcaaagcuugguaaagaugcccugugaaaguuuugcguguaucugagccucugaaacucaagaagacaucauauu
......................ccaguucauccaaacaccau........................................................................................................................................................................................................  19    0  JCK-3
......................ccaguucauccaaacaccau........................................................................................................................................................................................................  1     0  LT-3
......................ccaguucauccaaacaccau........................................................................................................................................................................................................  12    0  JCK-1
......................ccaguucauccaaacaccaA........................................................................................................................................................................................................  1     1  JCK-3
......................ccaguucauccaaacaccau........................................................................................................................................................................................................  39    0  JCK-2
......................ccaguucauccaaacaccau........................................................................................................................................................................................................  1     0  LT-1
......................ccaguucauccaaacaccaA........................................................................................................................................................................................................  3     1  JCK-1
......................ccaguucauccaaacaccau........................................................................................................................................................................................................  9     0  JT-3
......................ccaguucauccaaacaccau........................................................................................................................................................................................................  4     0  JT-2
......................ccaguucauccaaacCccaug.......................................................................................................................................................................................................  2     1  JCK-2
......................ccaguucauccaaaUaccaug.......................................................................................................................................................................................................  1     1  JCK-1
......................cUaguucauccaaacaccaug.......................................................................................................................................................................................................  5     1  JCK-1
......................ccaguucauccaaacaccGug.......................................................................................................................................................................................................  1     1  JCK-1
......................ccaguucauccaaacaAcaug.......................................................................................................................................................................................................  2     1  JCK-1
......................ccaguucauccaaacaAcaug.......................................................................................................................................................................................................  1     1  JCK-3
......................ccaguucauUcaaacaccaug.......................................................................................................................................................................................................  5     1  JCK-2
......................ccaguucauccaaacaccaug.......................................................................................................................................................................................................  4190  0  JCK-1
......................ccaguucauccCaacaccaug.......................................................................................................................................................................................................  2     1  LT-2
......................ccaguucauccaaaUaccaug.......................................................................................................................................................................................................  1     1  JT-2
......................ccaguucauccaaacaccaug.......................................................................................................................................................................................................  80    0  JT-1
......................ccaguuAauccaaacaccaug.......................................................................................................................................................................................................  1     1  JCK-1
......................ccaguucauccaaacacAaug.......................................................................................................................................................................................................  1     1  LT-2
......................ccaguucauccaaacaccaug.......................................................................................................................................................................................................  5156  0  JCK-3
......................ccaguucauccaaacaccaug.......................................................................................................................................................................................................  7056  0  JCK-2
......................ccCguucauccaaacaccaug.......................................................................................................................................................................................................  4     1  JCK-1
......................ccaguucauccaaacCccaug.......................................................................................................................................................................................................  2     1  LT-1
......................ccaguucauccaaUcaccaug.......................................................................................................................................................................................................  1     1  JCK-2
......................ccaguucauccaaCcaccaug.......................................................................................................................................................................................................  2     1  LT-3
......................ccaguucauccaaacaUcaug.......................................................................................................................................................................................................  2     1  JCK-2
......................ccaguucaCccaaacaccaug.......................................................................................................................................................................................................  2     1  JCK-1
......................ccaguucauccCaacaccaug.......................................................................................................................................................................................................  1     1  JCK-1
......................ccaguucauccaaacaccauU.......................................................................................................................................................................................................  2     1  JCK-2
......................ccaguucauccGaacaccaug.......................................................................................................................................................................................................  1     1  JCK-1
......................ccaguucaucGaaacaccaug.......................................................................................................................................................................................................  2     1  JCK-1
......................ccaguucauccNaacaccaug.......................................................................................................................................................................................................  9     1  JCK-1
......................ccaAuucauccaaacaccaug.......................................................................................................................................................................................................  2     1  JCK-1
......................ccaguucauccaaacaccauU.......................................................................................................................................................................................................  2     1  LT-1
......................ccaguucauccCaacaccaug.......................................................................................................................................................................................................  1     1  LCK-3
......................ccaAuucauccaaacaccaug.......................................................................................................................................................................................................  1     1  JT-3
......................ccaguucauccCaacaccaug.......................................................................................................................................................................................................  2     1  JCK-3
......................ccaguucauccaaCcaccaug.......................................................................................................................................................................................................  10    1  JCK-2
......................ccaguucaucUaaacaccaug.......................................................................................................................................................................................................  1     1  JCK-1
......................ccaguucauccaaacaccaug.......................................................................................................................................................................................................  932   0  LT-3
......................ccaguucauccaaacaccGug.......................................................................................................................................................................................................  1     1  JCK-3
......................ccaguucauccaaacaccauA.......................................................................................................................................................................................................  1     1  LT-3
......................ccaguucCuccaaacaccaug.......................................................................................................................................................................................................  9     1  JCK-2
......................ccaguucauccaaGcaccaug.......................................................................................................................................................................................................  2     1  LT-1
......................ccaguucauccaaacaccauA.......................................................................................................................................................................................................  4     1  JCK-2
......................ccaguucauccaaacaccaug.......................................................................................................................................................................................................  57    0  JT-3
......................ccaguucauccaaacaccauU.......................................................................................................................................................................................................  3     1  JCK-3
......................cUaguucauccaaacaccaug.......................................................................................................................................................................................................  2     1  JCK-3
......................ccaguucCuccaaacaccaug.......................................................................................................................................................................................................  1     1  LT-2
......................ccCguucauccaaacaccaug.......................................................................................................................................................................................................  1     1  LT-1
......................ccaguucauccaaacaccauC.......................................................................................................................................................................................................  1     1  LT-3
......................ccaguucauccaaacaccauA.......................................................................................................................................................................................................  1     1  JCK-1
......................ccaguucauccaaGcaccaug.......................................................................................................................................................................................................  1     1  JCK-2
......................ccaguucauccaaacaccCug.......................................................................................................................................................................................................  2     1  JCK-2
......................ccaguucauccaaacacAaug.......................................................................................................................................................................................................  1     1  JCK-3
......................ccaguucaucUaaacaccaug.......................................................................................................................................................................................................  3     1  JCK-2
......................ccaguucauccaaacCccaug.......................................................................................................................................................................................................  1     1  LT-3
......................ccaguucauccaaacaccaCg.......................................................................................................................................................................................................  2     1  JCK-2
......................ccaguucauccaCacaccaug.......................................................................................................................................................................................................  2     1  JCK-3
......................ccaguucauccaaacacGaug.......................................................................................................................................................................................................  1     1  JCK-1
......................ccaguucauccCaacaccaug.......................................................................................................................................................................................................  7     1  JCK-2
......................cNaguucauccaaacaccaug.......................................................................................................................................................................................................  8     1  JCK-1
......................ccaguGcauccaaacaccaug.......................................................................................................................................................................................................  1     1  JCK-2
......................ccaguucauccaaacaccaug.......................................................................................................................................................................................................  29    0  JT-2
......................ccaguucauccaaacaccaug.......................................................................................................................................................................................................  370   0  LCK-3
......................ccaguucCuccaaacaccaug.......................................................................................................................................................................................................  6     1  JCK-3
......................ccaguucauccaaacaccauU.......................................................................................................................................................................................................  3     1  LT-3
```

Mature **Star**

ucucgacaaucaaggaucuccaccaguucauccaaacaccaugaagaaaaugcauaguuuuuggagaagaugaugauggauaucuuugauccuccaauugcgucaauaagugaaggugaaauuucuucuccccucuugagcuuauugucaaugaaagucucaacuccccuaagacgcaaagcuugguaaagaugcccugugaaaguuuugcguguaucugagccucugaaacucaagaagacaucauauu			
......................ccagCucauccaaacaccaug	1	1	JCK-2
......................ccaguucaCccaaacaccaug	1	1	JCK-2
......................ccaguucauccaaacaccauN	1	1	JCK-1
......................ccaguucauccaaacacAaug	1	1	LT-1
......................ccaguucauccCaacaccaug	1	1	LT-1
......................ccaguucauccaaacaAcaug	1	1	JCK-2
......................ccaguucauccaaacaccauU	27	1	LT-2
......................cUaguucauccaaacaccaug	3	1	JCK-2
......................Ncaguucauccaaacaccaug	1	1	JCK-3
......................ccaguucauccaaacaccaug	1	0	LCK-2
......................ccaguucauccaaacaUcaug	2	1	JCK-3
......................ccaguucauccaaacaccaGg	1	1	JCK-3
......................ccagCucauccaaacaccaug	2	1	JCK-3
......................ccaguucauccaaacGccaug	1	1	JCK-3
......................ccaguucauccaaacaccaug	1097	0	LT-1
......................ccaguucauccaaacaAcaug	1	1	LT-1
......................ccCguucauccaaacaccaug	3	1	JCK-3
......................ccaguucauccCaacaccaug	1	1	LT-3
......................ccaguucaucUaaacaccaug	3	1	JCK-3
......................ccaguucauccaaacaccauA	1	1	JCK-3
......................ccaguucaucAaaacaccaug	1	1	JCK-3
......................ccaguucauccaaCcaccaug	6	1	JCK-3
......................ccaguucauccaaacaccauU	8	1	JCK-1
......................ccaguucauccaaacaccCug	2	1	JCK-1
......................ccaguucauccaaCcaccaug	1	1	LT-2
......................ccGguucauccaaacaccaug	2	1	JCK-2
......................ccCguucauccaaacaccaug	3	1	LT-2
......................ccaguucauccaaacaccauU	14	1	LCK-3
......................ccaguucauccaCacaccaug	12	1	JCK-2
......................ccaguCcauccaaacaccaug	3	1	JCK-3
......................ccaguucauccaaacCccaug	1	1	JCK-1
......................ccaguucCuccaaacaccaug	3	1	JCK-1
......................ccaguucauccaaaAaccaug	2	1	LT-1
......................ccaguuUauccaaacaccaug	1	1	JCK-2
......................ccaguucauccaaCcaccaug	8	1	JCK-1
......................ccaguucauccaaacaccCug	1	1	JCK-3
......................ccaguucauccaaacaccauC	3	1	JCK-1
......................ccaguucauAcaaacaccaug	1	1	JCK-1
......................ccaguucauccaGacaccaug	1	1	JCK-3
......................ccaguucauccaaaUaccaug	2	1	JCK-3
......................ccaguucauccaaaUaccaug	3	1	JCK-2
......................ccaguucauccaaacCccaug	1	1	JCK-3
......................ccaguucauccaaacaccNug	2	1	JCK-1
......................ccaguucauccaCacaccaug	1	1	LT-3
......................ccCguucauccaaacaccaug	1	1	LT-3
......................ccaguucaCccaaacaccaug	2	1	JCK-3
......................ccaguucauccaaacacUaug	1	1	JCK-2
......................ccaNuucauccaaacaccaug	5	1	JCK-1
......................ccaguucauccaaacaccaNg	9	1	JCK-1
......................cNaguucauccaaacaccaug	6	1	JCK-2
......................ccaguucauccaaacacAaug	2	1	JCK-2
......................ccaguucauccaaacaccaug	501	0	LT-2
......................ccCguucauccaaacaccaug	6	1	JCK-2
......................ccaguucauccaaacaUcaug	3	1	JCK-1
......................ccGguucauccaaacaccaug	1	1	JCK-3
......................ccaguucCuccaaacaccaug	1	1	LT-3
......................cNaguucauccaaacaccaug	5	1	JCK-3
......................ccaguucauccaaacaccauga	10	0	LCK-3
......................Ucaguucauccaaacaccauga	10	1	LT-2
......................ccaguucauccaaacaccaugG	2	1	JCK-1
......................ccaguucauccaaacaccauga	2	0	LT-2
......................ccaguucauccaaacaccauga	10	0	JCK-1
......................Ucaguucauccaaacaccauga	4	1	JCK-3
......................ccaguucauccaaacaccaugC	1	1	JCK-2
......................Ucaguucauccaaacaccauga	7	1	LCK-3
......................ccaguucauccaaacaccauga	20	0	JCK-2
......................Ucaguucauccaaacaccauga	4	1	LT-1
......................ccaguucauccaaacaccauga	6	0	LT-3
......................ccaguucauccaaacaccauUa	1	1	JCK-3
......................Ucaguucauccaaacaccauga	2	1	JCK-1

```
                      Mature                          Star
ucucgacaaucaaggaucuccaccaguucauccaaacaccaugaagaaaaugcauaguuuuuggagaagaugaugauggauaucuuugauccuccaauugcgucaauaagugaaggugaaauuucuucuccccucuugagcuuauugucaaugaaagucucaacucccccuaagacgcaaagcuugguaaagaugcccugugaaaguuuugcguguaucugagccucugaaacucaagaagacaucauauu
......................Ucaguucauccaaacaccauga...............................................................................................................................................................................    5    1    LT-3
......................Ucaguucauccaaacaccauga...............................................................................................................................................................................    2    1    JCK-2
......................ccaguucauccaaacaccauga...............................................................................................................................................................................    6    0    LT-1
......................ccaguucauccaaCcaccauga...............................................................................................................................................................................    1    1    JCK-2
......................ccaguucauccaaacaccauga...............................................................................................................................................................................    14   0    JCK-3
......................Ucaguucauccaaacaccaugaa..............................................................................................................................................................................    1    1    JCK-2
.......................caguucauccaaacacca..................................................................................................................................................................................    3    0    LT-2
.......................caguucauccaaacacca..................................................................................................................................................................................    3    0    JCK-1
.......................caguucauccaaacacca..................................................................................................................................................................................    2    0    LT-1
.......................caguucauccaaacacca..................................................................................................................................................................................    12   0    JT-1
.......................caguucCuccaaacacca..................................................................................................................................................................................    1    1    LT-2
.......................caguucauccaaacacca..................................................................................................................................................................................    7    0    LT-3
.......................caguucauccaaacaccau.................................................................................................................................................................................    2    0    LT-1
.......................caguucauccaaacaccau.................................................................................................................................................................................    2    0    JCK-2
.......................caguucauccaaacaccau.................................................................................................................................................................................    2    0    LT-2
.......................caguucauccaaacaccau.................................................................................................................................................................................    2    0    LT-3
.......................caguucauccaaacaccauC................................................................................................................................................................................    1    1    LT-1
.......................caguucCuccaaacaccaug................................................................................................................................................................................    1    1    JCK-1
.......................caguucauccaaacaccaug................................................................................................................................................................................    37   0    JT-1
.......................caguucauccaaacaccauU................................................................................................................................................................................    2    1    LT-1
.......................caguucauccaaacaccauU................................................................................................................................................................................    2    1    JCK-2
.......................caguucauccaaacaccaug................................................................................................................................................................................    27   0    LT-3
.......................caguucauccaaCcaccaug................................................................................................................................................................................    1    1    JCK-3
.......................caguucauccaaacaccaug................................................................................................................................................................................    63   0    JCK-2
.......................caguucauccaaacaccaug................................................................................................................................................................................    41   0    LT-1
.......................caguucauccaaCcaccaug................................................................................................................................................................................    1    1    JCK-2
.......................caguucauccaaacaccaug................................................................................................................................................................................    25   0    LCK-3
.......................caguucauccaaacaccaug................................................................................................................................................................................    49   0    JCK-3
.......................caguucauccaaacaccauU................................................................................................................................................................................    4    1    JCK-3
.......................caguucauccaaacaccauU................................................................................................................................................................................    4    1    LT-3
.......................caguucauccaaacaccaug................................................................................................................................................................................    38   0    JCK-1
.......................caguucauccaaacaccauU................................................................................................................................................................................    3    1    JCK-1
.......................caguucCuccaaacaccaug................................................................................................................................................................................    1    1    LT-2
.......................caguucauccaaacaccaug................................................................................................................................................................................    7    0    JT-3
.......................caguucauccaaacaccaug................................................................................................................................................................................    46   0    LT-2
.......................caguucauccaCacaccaug................................................................................................................................................................................    1    1    LT-2
.......................cCguucauccaaacaccaug................................................................................................................................................................................    1    1    JCK-3
.......................caguucauccaCacaccaug................................................................................................................................................................................    1    1    JCK-2
.......................caguucauccaaacaccaug................................................................................................................................................................................    3    0    JT-2
.......................caguucauccaaacaccauga...............................................................................................................................................................................    1    0    LT-1
.......................caguucauccaaacaccauCa...............................................................................................................................................................................    4    1    JCK-2
.......................caguucauccaaacaccauUa...............................................................................................................................................................................    4    1    LT-2
.......................caguucauccaaacaccauUa...............................................................................................................................................................................    1    1    LT-3
.......................caguucauccaaacaccaugUa..............................................................................................................................................................................    1    1    JCK-3
.......................caguucauccaaacaccaugUa..............................................................................................................................................................................    5    1    LT-2
.......................caguucauccaaacaccaugUa..............................................................................................................................................................................    1    1    JCK-1
........................aguucauccaaacaccau.................................................................................................................................................................................    1    0    JCK-2
........................aguucauccaaacaccaug................................................................................................................................................................................    8    0    JCK-3
........................aguucauccaaacaccaug................................................................................................................................................................................    10   0    JCK-2
........................aguucauccaaacaccaug................................................................................................................................................................................    10   0    LT-3
........................aguucauccaaacaccaug................................................................................................................................................................................    6    0    JCK-1
........................aguucauccaaacaccaug................................................................................................................................................................................    5    0    LCK-3
........................aguucauccaaacaccaug................................................................................................................................................................................    11   0    LT-1
........................aguucauccCaacaccaug................................................................................................................................................................................    1    1    LT-3
........................aguucauccaaacaccaug................................................................................................................................................................................    12   0    LT-2
...........................ucauccaaacaccaugUa..............................................................................................................................................................................    5    1    LT-3
.........................................................guuuuGggagaagaugau................................................................................................................................................................................    1    1    LT-3
..........................................................uuuGggagaagaugauga...............................................................................................................................................................................    1    1    JT-3
..........................................................uuuuggUgaagaugauga...............................................................................................................................................................................    1    1    LT-1
..........................................................uuuuggUgaagaugauga...............................................................................................................................................................................    8    1    JT-2
..........................................................uuuGggagaagaugauga...............................................................................................................................................................................    5    1    JT-2
..........................................................uuuuggUgaagaugauga...............................................................................................................................................................................    9    1    JT-3
...........................................................uuuggUgaagaugaugau..............................................................................................................................................................................    4    1    LT-2
............................................................uuggUgaagaugaugaug.............................................................................................................................................................................    1    1    LT-1
```
